# Analysis of gene expression profiles in two spinal cord injury models

**DOI:** 10.1186/s40001-022-00785-x

**Published:** 2022-08-23

**Authors:** Haifeng Yuan, Bi Zhang, Junchi Ma, Yufei Zhang, Yifan Tuo, Xusheng Li

**Affiliations:** 1 Department of Spinal Orthopedics, General Hospital of Ningxia Medical University, No. 804 Shengli Street, Xingqing District, Yinchuan, 750004 China; 2 Department of Anesthesia, Ningbo Medical Center Li Huili Hospital, Ningbo, 315046 China; 3 Department of Orthopaedics, Affiliated Hospital of Gansu College of Traditional Chinese Medicine, Lanzhou, 730099 China; 4 The third department of spine, Baoji Hospital of Traditional Chinese Medicine, Baoji, 721001 China; 5 Clinical Medicine, Ningxia Medical University, Yinchuan, 750004 China

**Keywords:** Spinal cord injury, RNA sequencing, Differentially expressed genes, Signaling pathway

## Abstract

**Objectives:**

To analyze the changes of gene expression at different timepoints after spinal cord injury (SCI) with tenth segment thoracic injury.

**Methods:**

Two SCI models, the complete paraplegia (H) and Allen’s strike (D) methods were applied to induce SCI in rats, and transcriptome sequencing was performed 1, 3, 7, 14, 56, and 70 days after SCI, respectively. Principal component analysis, differentially expressed gene analysis, and hierarchical clustering analysis were applied to analyze the differentially expressed genes (DEGs). Gene Ontology GO enrichment analysis, Kyoto Encyclopedia of Genes and Genomes enrichment analysis, and Gene Set Enrichment Analysis revealed the pathway of gene enrichment.

**Results:**

There were 1,907, 3,120, 3,728, 978, 2,319, and 3,798 DEGs in the complete paraplegia group and 2,380, 878, 1,543, 6,040, 1,945, and 3,850 DEGs in the Allen’s strike method group and after SCI at 1, 3, 7, 14, 56, and 70 days, respectively. The transcriptome contours of D1, H1, D3, and H14 were clustered with C; the H56, D56, H70, and D70 transcriptome contours were similar and clustered together. H3, D7, and H7 were clustered together, and D14 was clustered separately. The transcriptome differences of the two SCI models were mainly concentrated during the first 2 weeks after SCI. The DEGs after SCI in the complete paraplegia group were more concentrated. Most of the early transcriptional regulation stabilized within 2 weeks after injury.

**Conclusions:**

There were DEGs between the two SCI models. Through the gene changes and pathway enrichment of the entire time period after SCI, the molecular mechanism of SCI repair was revealed in depth, which provided a reference for SCI treatment in the future.

## Introduction

Spinal cord injury (SCI) is a destructive neurological and pathological state that causes major motor, sensory, and autonomic dysfunctions, often with lifelong consequences [1–3]. According to statistics, the global annual SCI incidence is between 250,000 and 500,000 [4]. As well as the direct consequences of the loss of sensory, motor, and autonomic nervous system functions, secondary processes in the injured area may exacerbate the injury, including chronic pain, muscle wasting, pressure sores, and urinary tract infections [5]. Consequently, the pathological and physiological changes are divided into two stages: primary and secondary injury [6]. When the spinal cord is subjected to injury, tearing, or compression due to external forces or infarction due to vascular injury, the direct and immediate physical disruption represents the primary injury [7]. Secondary injury is a delayed and prolonged pathological stage caused by a primary injury, which exacerbates spinal cord tissue injury through the occurrence of a series of biological events [8, 9]. Ultimately, it may lead to motor function loss, various organ dysfunctions, and chronic neuropathic pain [10]. The complex and inconsistent pathophysiological consequences of SCI have led to an insufficient understanding, which has become the main reason for treatment failure. SCI reduces the quality of life of patients and causes great emotional and economic burdens [6]. However, the treatment methods and effects of SCI remain limited, and more effective treatment methods are urgently required.

Currently, many studies are also committed to developing neuroprotective and nerve regeneration therapies to promote neuronal recovery. Although this treatment has achieved varying degrees of success, the outcome remains uncertain due to the complex healing mechanisms involved. Therefore, a more intensive investigation of the mechanism and molecular events of SCI and its secondary damage is a significant approach to developing SCI treatment plans [11, 12]. Normal spinal cord physiology involves the interaction between many cell types, but after spinal injury, these multicellular interactions are interrupted, resulting in impaired spinal recovery [13]. Various animal studies have shown that the current SCI treatment has achieved certain results in reducing neuroinflammation, enhancing myelination, promoting axon growth, and reducing the size of cavities [14]. However, the current treatment strategies still cannot completely overcome the damaging effects of SCI.

The ultimate goal of SCI research is to develop and provide effective repair and treatment strategies for clinical development [15]. Previous studies usually directly selected a single gene to detect and analyze the regulatory pathway. Second-generation high-throughput sequencing is realized by extracting all transcribed RNA from samples and then reverse transcribing it into cDNA [6]. On this basis, the fragments were overlapped and assembled to obtain transcripts to observe the changes after transcription and gene fusion and expression [16]. Using the second-generation sequencing technology to dynamically monitor the transcriptome change of rats to determine and understand the entire process of SCI pathophysiology and the sequence of events during and after injury, this is conducive to the design of interventions suitable for SCI [3].

Therefore, the use of RNA-seq (RNA-sequencing) technology to efficiently analyze the sequence of events during and after injury in different injury models enables a comprehensive understanding of the mechanism of SCI and its secondary injury, thereby providing data for the further study of the molecular mechanism of SCI, and providing strategies for the recovery and treatment of neurological function after severe central nervous system injury in the future. The results of the present study provided a novel insight into the mechanisms underlying SCI.

## Materials and methods

### Experimental animals

A total of 39 specific-pathogen-free 8-week-old adult female Sprague–Dawley rats weighing 200–220 g were purchased from the Shanghai SLAC Laboratory Animal Co. Ltd. (Shanghai, China). Animal care in the surgical procedures and post-operation were in accordance with the Regulations for the Administration of Affairs Concerning Experimental Animals and Guidelines and Policies for Rodent Survival Surgery provided by the Animal Care and Use Committees of Ningxia Medical University General Hospital.

### SCI model establishment

The 39 adult female rats were randomly assigned to a control group (C, 3 rats) and one of two SCI groups: complete paraplegia (H, 18 rats) and Allen's strike (D, 18 rats) groups. The SCI models were established after 3 days of adaptive feeding. The mice were anesthetized by intraperitoneal injection of 4% chloral hydrate. The surgical area was shaved and disinfected with 70% ethanol and betadine. A 5 cm long incision was made centered on T10, the spinous processes and lamina of the thoracic area (T9–T11) were separated and exposed, the spinous processes and lamina were removed, the spinal cord was fully exposed, and an incomplete cross-cut was made with ophthalmic scissors or scalpel for the group H. In the group D, a 5 g hammer was fixed at a height of 16 cm in a smashing device and was allowed to fall freely to impact the T9–T11 spinal cord tissue [17]. All rats were kept warm after the operation. After awakening from the anesthesia, they were transferred to the animal room and fed normally. The animals received 20,000 IU of penicillin by intraperitoneal injection once a day for the first 3 day post-operation, and bladder urine was squeezed twice a day manually. In case of mortality during the experiment, the process of generating the particular model was repeated to supplement the specific time subgroup. In the two experimental groups, three animals were euthanized on days 1, 3, 7, 14, 56, and 70 after SCI, with three rats in the control group euthanized on day 70.

### RNA-Seq technology and bioinformatics analysis

Rats were euthanized 1, 3, 7, 14, 56, and 70 days after injury, and a 2 cm wound tissue sample was taken from the injury site for RNA analysis. Total RNA was extracted with TRIzol reagent (Invitrogen, Burlington, ON, Canada) [17]. RNA integrity was assessed using the RNA Nano 6000 Assay Kit of the Bioanalyzer 2100 system (Agilent Technologies, Santa Clara, CA, USA) [18]. Quantified samples were sent for RNA-Seq analysis at Novogene Corporation (Beijing, China) [19].

### Differential expression analysis

FPKM (Fragments Per Kilobase Million) were considered to estimate the gene expression level [20]. DESeq2 was applied to determine differential expression in the digital gene expression data. An adjusted *P* < 0.05 was defined as differentially expressed [21]. In addition to FPKM hierarchical clustering analysis of differentially expressed genes (DEGs), we further analyzed the subclusters based on log2 (ratios) of their gene expression level relative to that of the control group. The log2 (ratios) in the D and the Hgroups of  ≥ 1 or  ≤ − 1 was applied as a cutoff for subcluster analysis. The clustering algorithm divided the DEGs with similar gene expression trends into several subclusters.

### GO and KEGG enrichment analysis of DEGs

Gene Ontology (GO) enrichment analysis and Kyoto Encyclopedia of Genes and Genomes (KEGG) (http://www.genome.jp/kegg/) of DEGs were implemented using the clusterProfiler R package, in which gene length bias was corrected. *P* < 0.05 were considered significantly enriched by DEGs.

### Gene set enrichment analysis

Gene Set Enrichment Analysis (GSEA) is a pre-defined gene set that can show a significant consistent difference between two biological states. The genes were ranked according to the degree of differential expression in the two samples, and then the predefined gene set was tested to determine whether they were enriched at the top or bottom of the list. GSEA can distinguish between subtle expression changes. We used the local version of the GSEA analysis tool http://www.broadinstitute.org/gsea/index.jsp, and the GO and KEGG data sets were used independently for GSEA.

## Results

### Identification and distribution of expressed transcripts in rat spinal cord

**Fig. 1:**
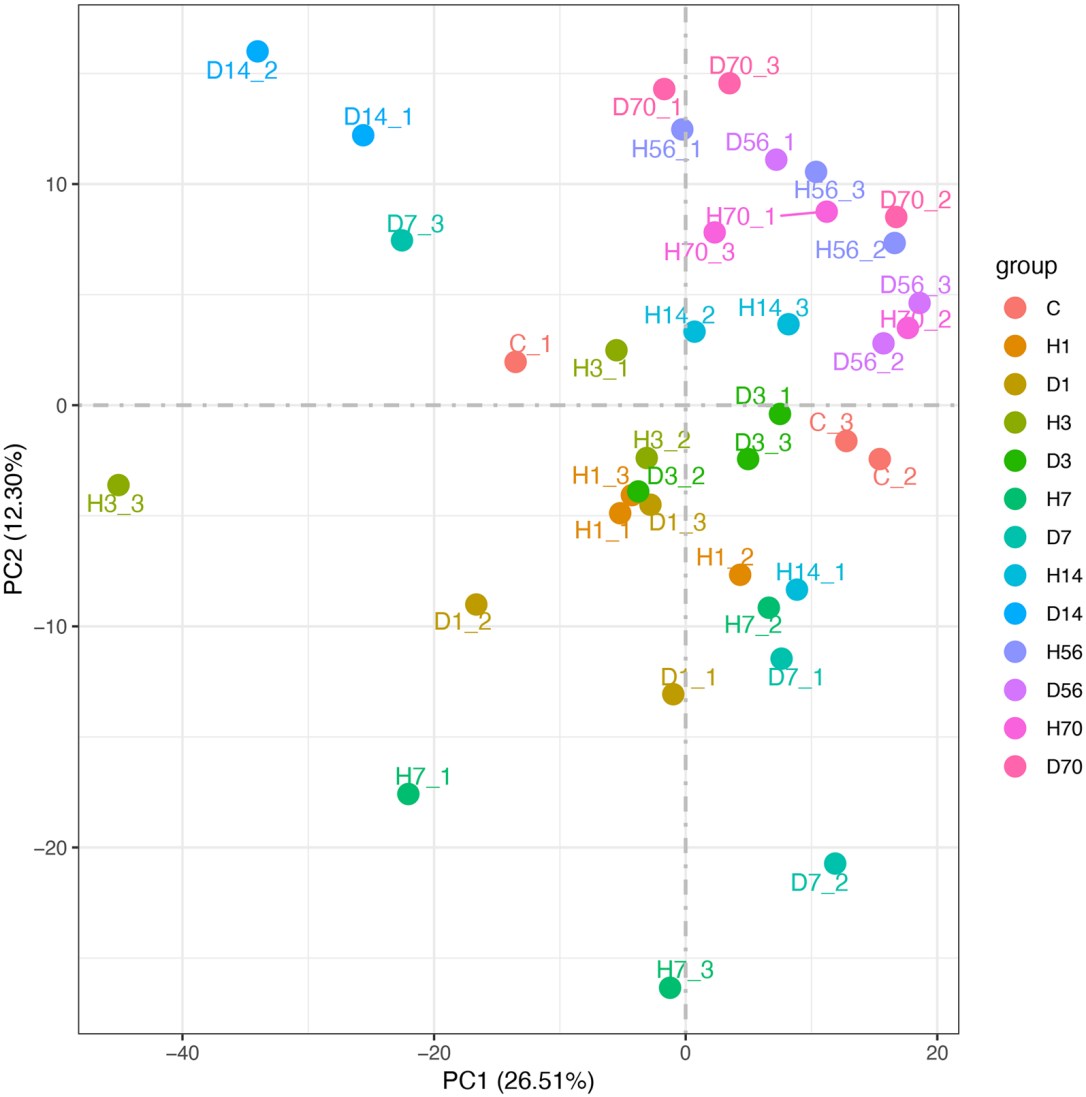
PCA analysis for the complete paraplegia (H) and Allen’s strike (D) groups. PCA analysis was applied to evaluate the differences between groups and the duplication of samples within groups. Control group (C), complete paraplegia group (H1, H3, H7, H14, H56, and H70), and Allen’s strike method group (D1, D3, D7, D14, D56, and D70). PCA, principal component analysis

For the high quality assessment of sequencing data, 39 cDNA libraries were established, including the group C, complete paraplegia group (H1, H3, H7, H14, H56, and H70), and Allen's strike method group (D1, D3, D7, D14, D56, and D70). RNA-Seq produced 39,435,984–52,386,642 raw reads for each sample. After filtering out the low-quality reads, there were 38,663,500–50,797,750 clean reads, with the Q20 97.46–98.58% and the Q30 92.76–95.66%. To identify the source of variation within the original data and evaluate the difference between groups and the duplication of samples within groups, PCA (Principal component analysis) was conducted. As shown in Fig. 1, PC1 and PC2 was 26.51% and 12.30%, respectively. The samples within the groups were relatively concentrated, whereas the samples between the groups were dispersed, and the degree of dispersion was related to the time after SCI. The difference between samples in the complete paraplegia group gradually increased with time, attained a peak at 7 days after SCI, and there was little difference between samples in this group at 14, 56, and 70 days after SCI. The Allen’s strike method group displayed considerable changes immediately on the first day after SCI, D3 was closer to group C, whereas D7 and D14 were far away from group C, which was quite different from the group C. Consistent with the complete paraplegia group, there was little difference between 56 and 70 days after SCI, and H56, H70, D56, and D70 were clustered together.

### Effects of different SCI methods on gene expression

**Fig. 2:**
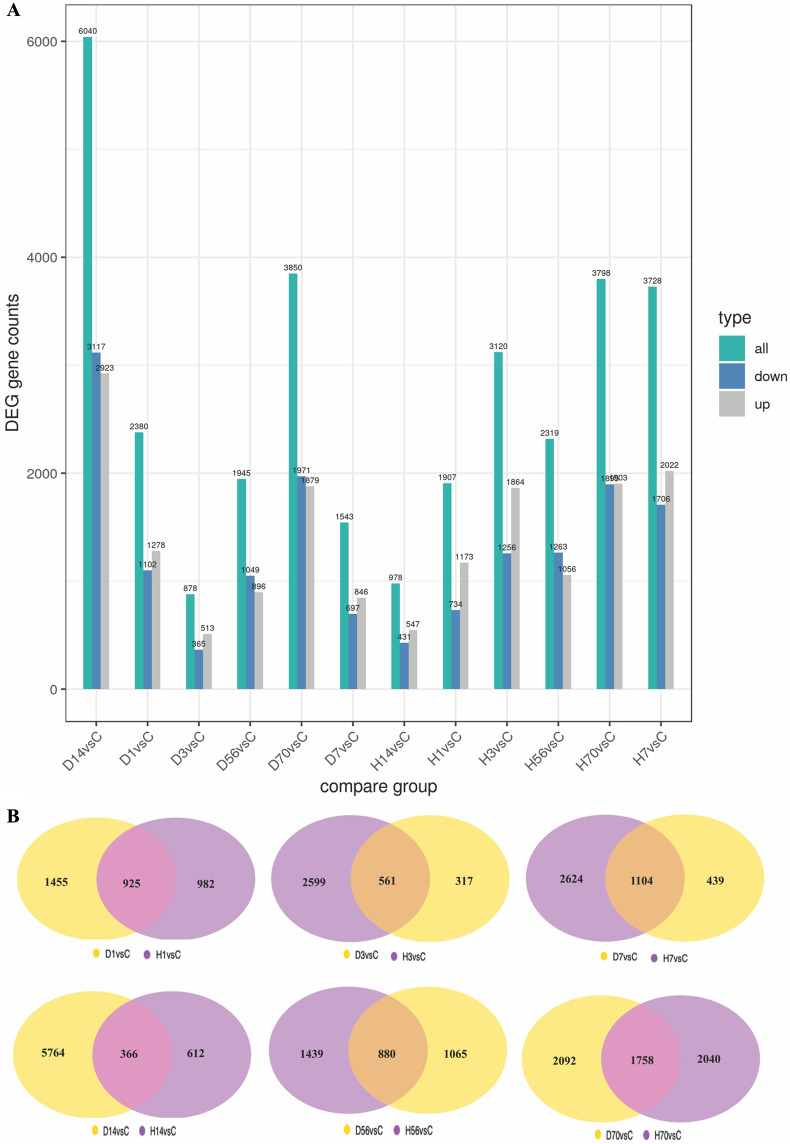
Statistical analysis of the differential genes number. (A) Statistical histogram of the number of differential genes in the complete paraplegia (H) and Allen’s strike (D) groups. (B) Differential gene Venn plot

RPKM and DESeq were used to analyze the gene expression level and differential expression profiles, respectively. The results are shown in Fig. 2A, as compared to the control group, there were 2,380, 878, 1,543, 6,040, 1,945, and 3,850 DEGs in the Allen’s strike method group and 1,907, 3,120, 3,728, 978, 2,319, 3,798 DEGs in the complete paraplegia group after SCI on days 1, 3, 7, 14, 56, and 70, respectively. The most genetic changes occurred on the day 14 after SCI in the Allen’s strike method group, including 3,117 upregulated and 2,923 downregulated genes. The most genetic changes occurred on day 7 after SCI in the complete paraplegia group, including 1,706 upregulated and 2,022 downregulated genes in the H7 group. The results of the Venn plot, shown in Fig. 2B, indicated that the number of common differential genes between the two SCI models was the greatest on day 70 after SCI, followed by day 7, whereas the least number of common differential genes between the two SCI models was on day 14, followed by day 3 after SCI.

### Cluster analysis of DEGs

**Fig. 3:**
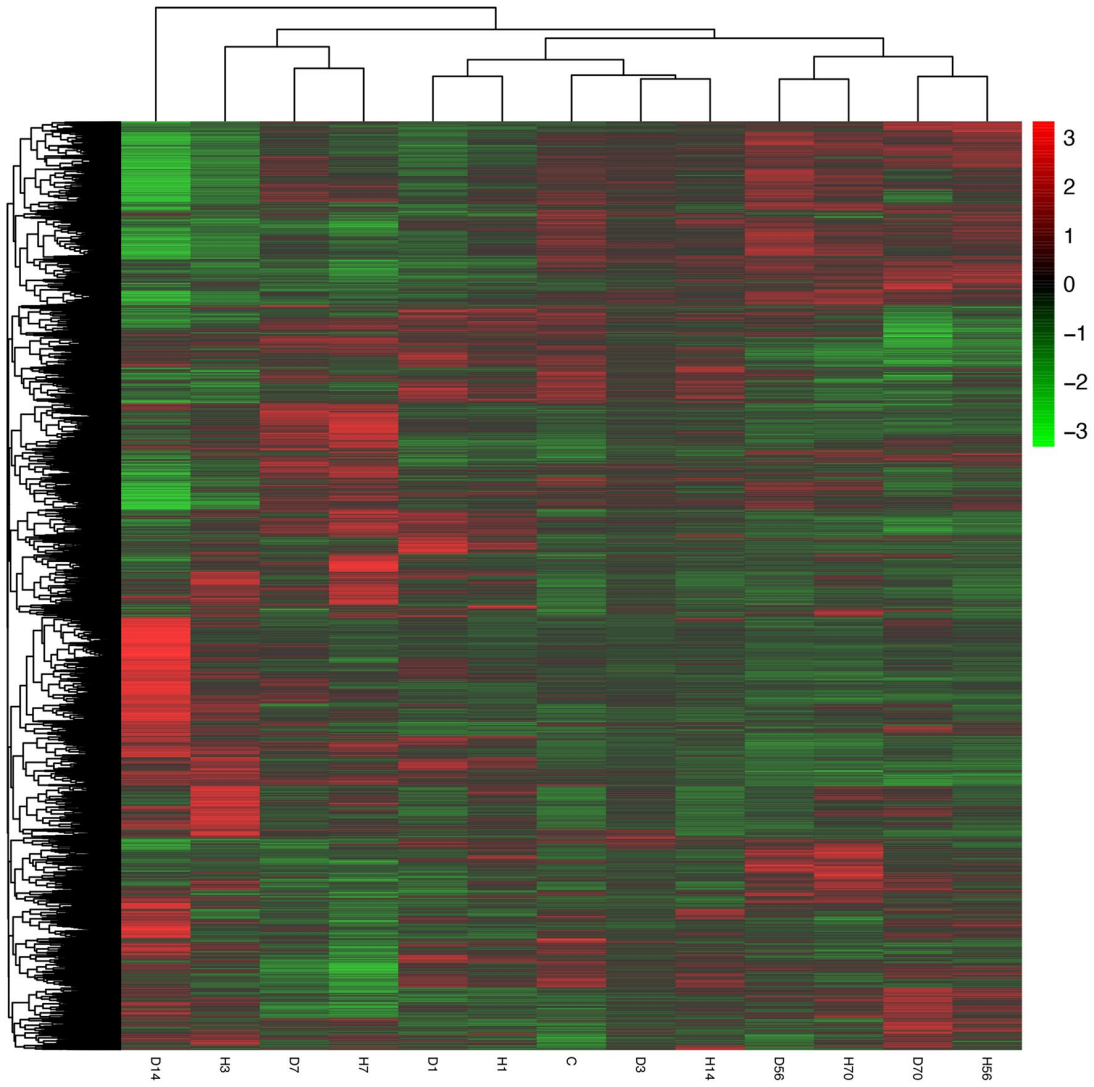
Hierarchical cluster analysis of DEGs. The DEGs in the different groups were analyzed using FPKM hierarchical cluster analysis. Corrected *P* value of 0.05 and absolute fold change of two were set as the threshold for significant differential expression. The color key indicates reads per kilobase per million reads normalized log2 transformed counts. The red color represents high expression. The green color represents low expression

Heat map and hierarchical clustering were used to determine the gene expression patterns in the rat transcriptome at different times after SCI in different SCI models. Genes with similar expression patterns may have similar functions or may participate in the same metabolic process or cellular pathway. In this study, FPKM was applied for hierarchical cluster analysis of transcriptome abundance, with the results shown in Fig. 3. D1, H1, D3, and H14 were clustered with C; H56, D56, H70, and D70 transcriptome contours were similar and clustered together. H3, D7, and H7 were clustered together, whereas D14 was clustered separately.

### GO enrichment analysis of DEGs

**Fig. 4:**
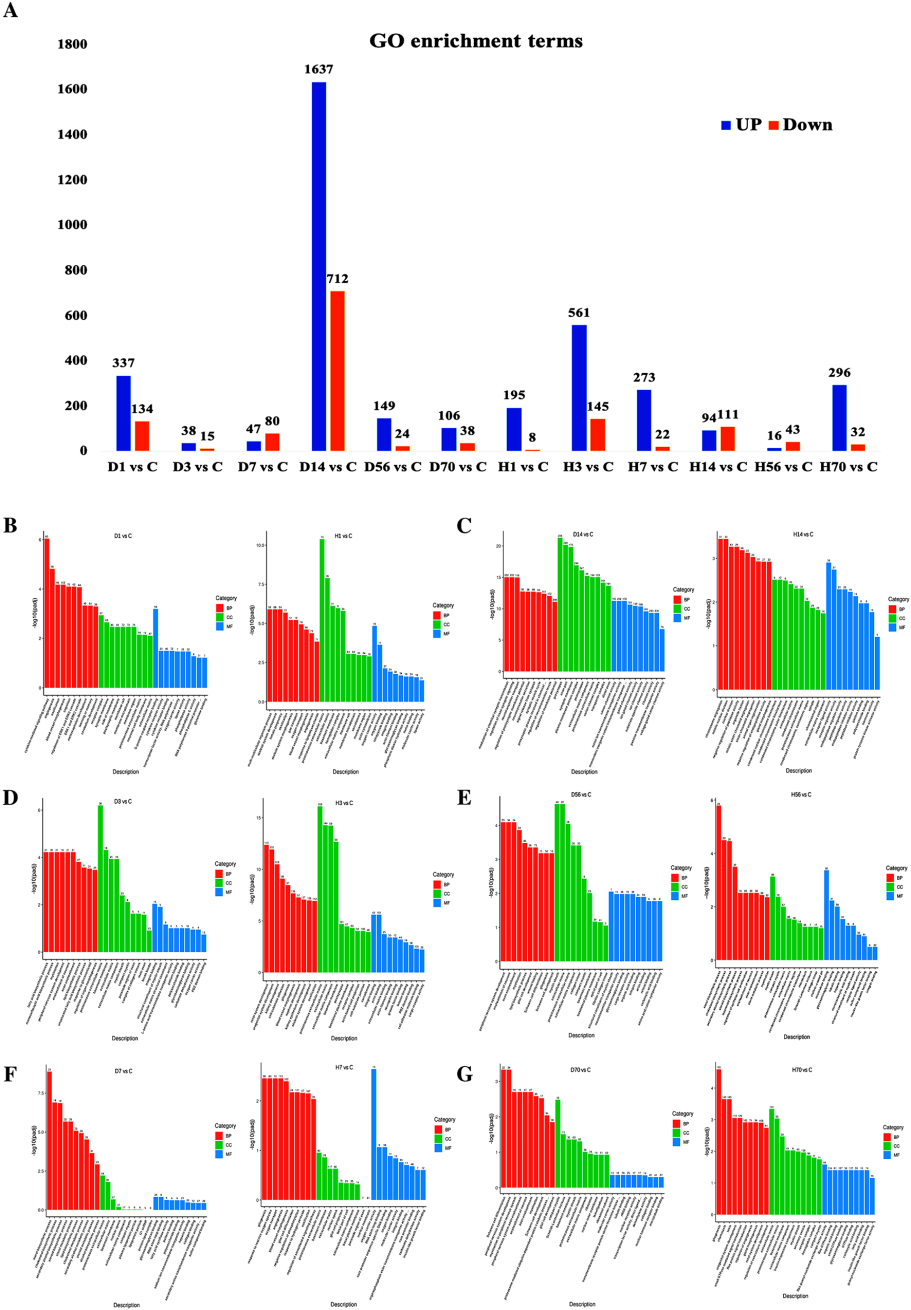
GO enrichment analysis of DEGs. **A** Number of GO terms enriched by upregulated DEGs and downregulated DEGs compared with the control group. **B**–**G** 10 richest GO terms of Molecular Function (MF), Biological Process (BP), and Cellular Component (CC) on days 1, 3, 7, 14, 56, 70 after spinal cord injury of the Allen’s strike (left) and complete paraplegia (right) groups. Note: the abscissa in the figure is the GO term, while the ordinate is the significance level of GO term enrichment, which is represented by −log_10_ (Padj), and different colors represent different functional classifications

Compared with the control group, the number of GO terms significantly enriched by upregulated and downregulated DEGs is shown in Fig. 4A. The 10 richest GO terms of molecular function, biological process, and cellular component on days 1, 3, 7, 14, 56, and 70 after SCI in the Allen’s strike and complete paraplegia groups are listed in Fig. 4B–G.

### KEGG enrichment analysis of DEGs

**Fig. 5:**
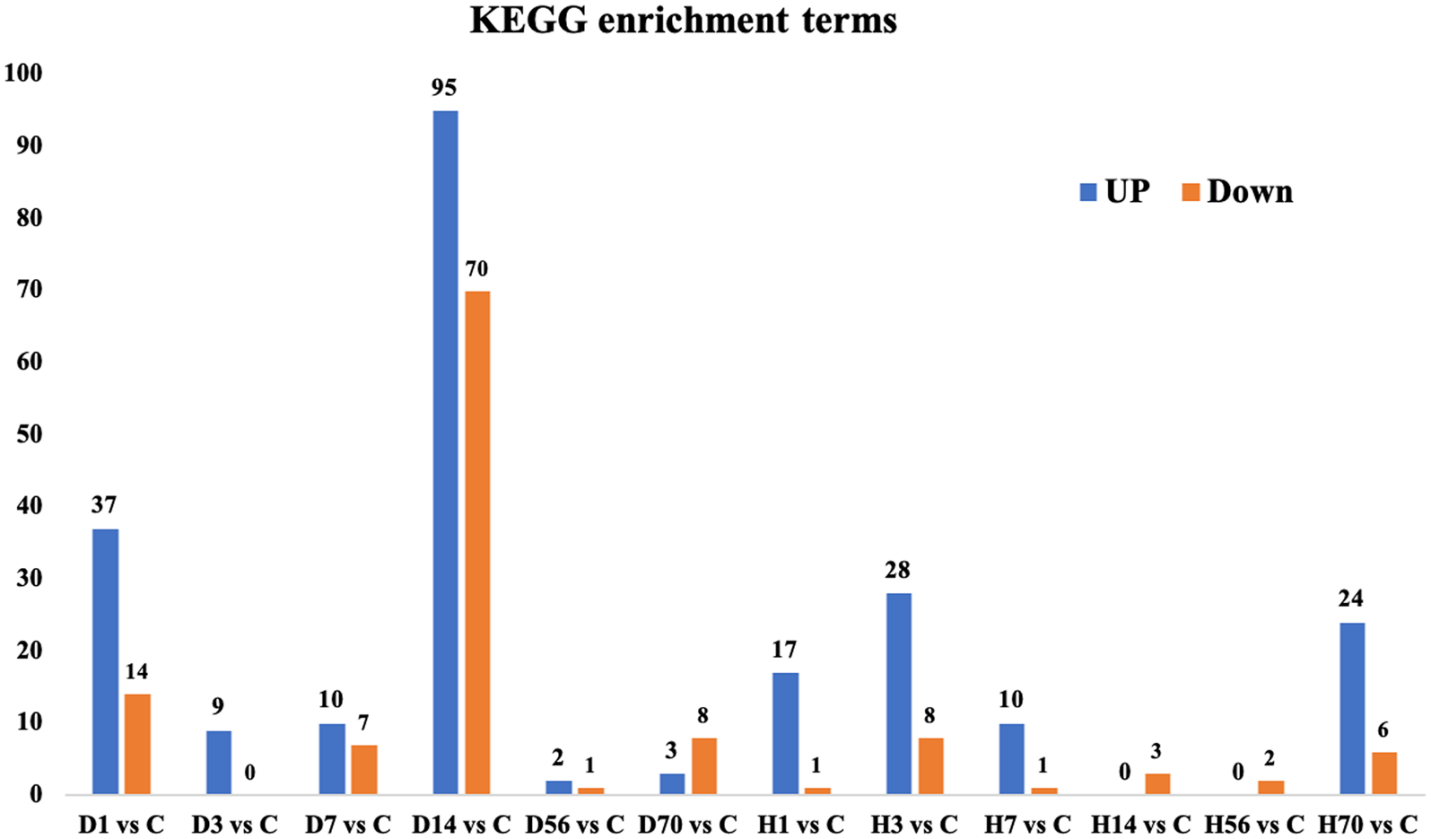
KEGG enrichment analysis of DEGs. The number of KEGG terms enriched by upregulated DEGs and downregulated DEGs compared with the control group

Compared with the control group, the number of KEGG terms significantly enriched by upregulated and downregulated DEGs is shown in Fig. 5. The top 10 KEGG terms of the enrichment analysis results of the DEGs are listed in Table1.

**Table 1:** Top 10 KEGG terms across all timepoints post-injury

Timepoint (days)	Sample	Differential expressed genes	Top 10 KEGG terms enrichment
1	D1 vs C	UP	Measles, TNF signaling pathway, Hepatitis C, African trypanosomiasis, JAK–STAT signaling pathway, Kaposi sarcoma-associated herpesvirus infection, Viral protein interaction with cytokine and cytokine receptor, IL-17 signaling pathway, NF-kappa B signaling pathway, cytokine–cytokine receptor interaction
		DOWN	Neuroactive ligand–receptor interaction, Other glycan degradation, Nicotine addiction, Morphine addiction, Glutamatergic synapse, Calcium signaling pathway, Circadian entrainment, Lysosome, Spinocerebellar ataxia, Oxytocin signaling pathway
	H1 vs C	UP	ECM–receptor interaction, Malaria, African trypanosomiasis, PI3K–Akt signaling pathway, Small cell lung cancer, Hypertrophic cardiomyopathy (HCM), MAPK signaling pathway, Dilated cardiomyopathy (DCM), Focal adhesion, Thyroid cancer
		DOWN	Other glycan degradation
3	D3 vs C	UP	ECM–receptor interaction, Focal adhesion, PI3K–Akt signaling pathway, Protein digestion and absorption, Small cell lung cancer, Arrhythmogenic right ventricular cardiomyopathy (ARVC), Hypertrophic cardiomyopathy (HCM), Hippo signaling pathway—multiple species, MAPK signaling pathway
		DOWN	/
	H3 vs C	UP	ECM–receptor interaction, PI3K–Akt signaling pathway, Focal adhesion, Hippo signaling pathway—multiple species, Small cell lung cancer, Malaria, Proteoglycans in cancer, Protein digestion and absorption, MAPK signaling pathway, African trypanosomiasis
		DOWN	Neuroactive ligand–receptor interaction, Retrograde endocannabinoid signaling, Morphine addiction, Gastric acid secretion, Ether lipid metabolism, GABAergic synapse, Glutamatergic synapse, Nicotine addiction
7	D7 vs C	UP	ECM–receptor interaction, Small cell lung cancer, PI3K–Akt signaling pathway, Transcriptional misregulation in cancer, FoxO signaling pathway, Chronic myeloid leukemia, p53 signaling pathway, Hippo signaling pathway—multiple species, Melanoma, Focal adhesion
		DOWN	Steroid biosynthesis, Terpenoid backbone biosynthesis, Fatty acid metabolism, Biosynthesis of unsaturated fatty acids, Neuroactive ligand–receptor interaction, Morphine addiction, Butanoate metabolism
	H7 vs C	UP	Malaria, PI3K–Akt signaling pathway, Hippo signaling pathway—multiple species, Small cell lung cancer, ECM–receptor interaction, Circadian rhythm, Chronic myeloid leukemia, Focal adhesion, African trypanosomiasis, Transcriptional misregulation in cancer
		DOWN	Steroid biosynthesis
14	D14 vs C	UP	Human papillomavirus infection, Epstein–Barr virus infection, ECM–receptor interaction, NOD-like receptor signaling pathway, NF-kappa B signaling pathway, Toxoplasmosis, Lysosome, Influenza A, Focal adhesion, Osteoclast differentiation
		DOWN	Retrograde endocannabinoid signaling, Parkinson disease, Glutamatergic synapse, Huntington disease, Alzheimer disease, Oxidative phosphorylation, GABAergic synapse, Thermogenesis, Neuroactive ligand–receptor interaction, Dopaminergic synapse
	H14 vs C	UP	–
		DOWN	Cell cycle, Progesterone-mediated oocyte maturation, Oocyte meiosis
56	D56 vs C	UP	Axon guidance, Nitrogen metabolism
		DOWN	Complement and coagulation cascades
	H56 vs C	UP	–
		DOWN	Steroid biosynthesis, Terpenoid backbone biosynthesis
70	D70 vs C	UP	ECM–receptor interaction, PI3K–Akt signaling pathway, Focal adhesion
		DOWN	Ribosome, Parkinson disease, Oxidative phosphorylation, Alzheimer disease, Non-alcoholic fatty liver disease (NAFLD), Huntington disease, Thermogenesis, Spliceosome
	H70 vs C	UP	ECM–receptor interaction, Rap1 signaling pathway, Focal adhesion, Hippo signaling pathway—multiple species, Circadian entrainment, PI3K–Akt signaling pathway, Aldosterone synthesis and secretion, Glucagon signaling pathway, Insulin resistance, African trypanosomiasis
		DOWN	Oxidative phosphorylation, Proteasome, Lysosome, Alzheimer disease, Parkinson disease

Top 10 KEGG terms enriched by upregulated DEGs and downregulated DEGs compared with the control group

### GSEA of KEGG

**Fig. 6:**
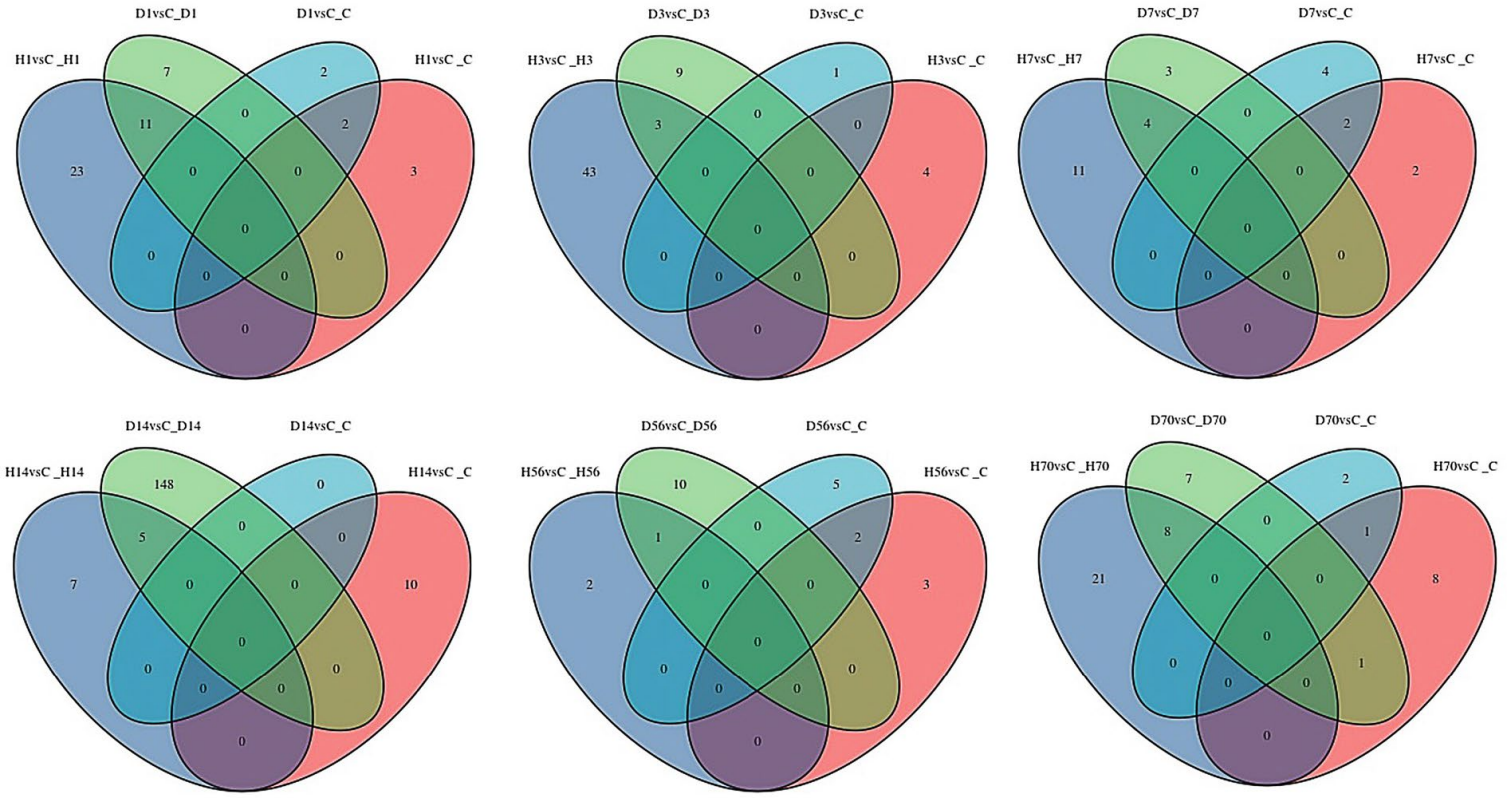
Venn plot of the common and unique KEGG pathway of gene sets. Venn plot of the common and unique KEGG pathway of gene sets in the complete paraplegia (H) and Allen’s strike (D) groups

To further investigate the changes of the transcriptome level after SCI, we analyzed the gene set enrichment of the complete paraplegia and Allen’s strike groups across all timepoints after SCI to investigate the transcriptome changes caused by SCI. Figure 6 shows the common and unique KEGG pathway of gene sets of the complete paraplegia and Allen’s strike groups. The common KEGG pathway of gene sets in the group H and group D was the most on days 1 and 70 after SCI. The unique KEGG pathway of gene sets in the complete paraplegia and Allen’s strike groups was the most on days 3 and 14 after SCI. Common and unique KEGG pathway items are shown in Table 2.

**Table 2:** Common gene sets of complete paraplegia group (H) and Allen’s strike group (D) across all timepoints post-injury

Timepoint (days)	Common upregulated gene sets	Common downregulated gene sets
1	L_17_SIGNALING_PATHWAY(RNO04657), SELENOCOMPOUND_METABOLISM(RNO00450), NON_SMALL_CELL_LUNG_CANCER(RNO05223), MELANOMA(RNO05218), BLADDER_CANCER(RNO05219), EGFR_TYROSINE_KINASE_INHIBITOR_RESISTANCE(RNO01521), ADIPOCYTOKINE_SIGNALING_PATHWAY(RNO04920), SPHINGOLIPID_SIGNALING_PATHWAY(RNO04071), MAPK_SIGNALING_PATHWAY(RNO04010), ESTROGEN_SIGNALING_PATHWAY(RNO04915), RAS_SIGNALING_PATHWAY(RNO04014)	STEROID_HORMONE_BIOSYNTHESIS(RNO00140), OOCYTE_MEIOSIS(RNO04114)
3	LYSINE_DEGRADATION(RNO00310), RENIN_ANGIOTENSIN_SYSTEM(RNO04614), AMPK_SIGNALING_PATHWAY(RNO04152)	–
7	BASAL_TRANSCRIPTION_FACTORS(RNO03022), RIBOSOME_BIOGENESIS_IN_EUKARYOTES(RNO03008), RNA_DEGRADATION(RNO03018), MRNA_SURVEILLANCE_PATHWAY(RNO03015)	STEROID_BIOSYNTHESIS(RNO00100), SEROTONERGIC_SYNAPSE(RNO04726)
14	PRIMARY_BILE_ACID_BIOSYNTHESIS(RNO00120), DRUG_METABOLISM___CYTOCHROME_P450(RNO00982), INSULIN_RESISTANCE(RNO04931), LYSINE_DEGRADATION(RNO00310), CHEMICAL_CARCINOGENESIS(RNO05204)	
56	STARCH_AND_SUCROSE_METABOLISM(RNO00500)	STEROID_BIOSYNTHESIS(RNO00100), SNARE_INTERACTIONS_IN_VESICULAR_TRANSPORT(RNO04130)
70	LYSINE_DEGRADATION(RNO00310), CIRCADIAN_RHYTHM(RNO04710), PHENYLALANINE_METABOLISM(RNO00360), LONGEVITY_REGULATING_PATHWAY___MULTIPLE_SPECIES(RNO04213) ABC_TRANSPORTERS(RNO02010), TYROSINE_METABOLISM(RNO00350), AMPK_SIGNALING_PATHWAY(RNO04152), RENIN_SECRETION(RNO04924), PURINE_METABOLISM(RNO00230)	FAT_DIGESTION_AND_ABSORPTION(RNO04975)

## Discussion

In recent years, research has focused on analyzing the complex pathological process and molecular mechanism of SCI to determine the impact of microenvironmental changes on SCI repair [6]. Currently, no completely restorative treatments for SCI are available [22, 23]. The present study applied the RNA-Seq technique to investigate the differences of transcriptome levels at different timepoints after SCI in different SCI models to investigate molecular mechanisms underlying SCI by bioinformatic methods. Unlike most early studies that mainly examined acute and subacute events, we extended the time range of the study to 10 weeks after injury to detect the changes of gene expression and related signal pathways during the entire repair process after SCI.

The results of the present study showed that the two SCI methods were quite different within 1–14 days, whereas they were relatively similar at 56 and 70 days. The difference between samples in the complete paraplegia group gradually increased with time, reached a peak at 7 days after SCI, while there was little difference between samples in this group at 14, 56, and 70 days after SCI. The Allen’s strike method group displayed considerable changes immediately on the first day after SCI, while D3 was closer to group C, but D7 and D14 were far away from group C, and was thus quite different from group C. Consistent with the complete paraplegia group, there was little difference between 56 and 70 days after SCI, and H56, H70, D56, and D70 were also gathered together. Consistent results were found in the heat map and hierarchical clustering, with D1, H1, D3, and H14 clustered with C; H56, D56, H70, and D70 transcriptome contours were similar and clustered together. H3, D7, and H7 were clustered together, whereas D14 was clustered separately. The transcriptome difference between the two SCI models was the largest on days and 14 after SCI.

Siebert et al. [24] demonstrated that there is a strong regenerative response during the early stages of SCI. In the present study, on the first day after SCI, DEGs were significantly enriched in “angiogenesis”, “vascular morphogenesis”, “vascular process in the circulatory system”, “wound healing”, “response to hydrogen peroxide”, and other signal pathways related to vascular regeneration. Previous studies have well-established that together with peripheral neutrophils, local microglia and monocytes migrate into the injury site and initiate an innate immune response at 1 day post-injury [25]. By 3–4 day post-injury, when active astrocytes, microglia, and oligodendrocyte progenitor cells are activated, a large degree of glial hyperplasia will appear. We found that “fatty acid biosynthetic process”, “myelination”, “peripheral nervous system development”, “ensheathment of neurons”, “axon ensheathment”, “lipid biosynthetic process”, “unsaturated fatty acid biosynthetic process”, “response to glucocorticoid” and related biological processes were significantly enriched on day 3 after SCI. At 7 day post-injury, “sterol metabolic process”, “lipid storage”, “sterol biosynthetic process”, “secondary alcohol biosynthetic process”, “regulation of lipid storage”, “cholesterol biosynthetic process”, “fatty acid biosynthetic process”, and related biological processes were significantly enriched. Previous studies have revealed that accumulating macrophages and fibroblasts start to form fibrotic scar, which is surrounded by a previously formed astroglia scar at 7 day post-injury [25–27]. Unlike previous studies that showed that revascularization and angiogenesis occurred during the first 7 days [28], the SCI induced angiogenesis and revascularization already on the first day. As consistent with previous studies, the genes involved in “blood coagulation” were up-regulated to a high level from days 1 to 14 [29]. Previous studies have shown that cell proliferation and tissue replacement occurs 2–10 days after injury to repair and regenerate tissue [3]. The cells involved in this stage include endothelial progenitor cells, glial and neural progenitor cells, inflammatory cells, fibroblasts, and scar-forming astrocytes [30]. Previous studies have determined that “the activation of the complement system”, “the induction of immune responses”, “adaptive immune responses”, and “the production of antibodies” based on GO functional analysis are the most significant up-regulated biological processes of various immune responses during acute SCI [31, 32]. In the present study, the differences between the two SCI models were concentrated during days 3–14 after SCI. At 14 day post-injury, “regulation of trans-synaptic signaling”, “neurotransmitter transport”, “modulation of chemical synaptic transmission”, “regulation of post-synaptic membrane potential”, “neurotransmitter secretion”, “signal release from synapse”, and related biological processes were significantly enriched in D14. However, in H14, more “chromosome segregation”, “negative regulation of peptidase activity”, “organelle fission”, “nuclear division”, “sister chromatid segregation”, “mitotic nuclear division”, “epithelial cell proliferation”, “mitotic sister chromatid segregation”, and related biological processes were significantly enriched.

At 56 and 70 day post-injury, the transcriptome characteristics of the two SCI models were relatively similar, while the relevant differential genes that were significantly enriched were in “glial cell development”, “epithelial cell proliferation”, “urogenital system development”, “gliogenesis”, “glial cell differentiation”, “regulation of angiogenesis”, “renal system development”, “glomerulus development”, “nephron development”, and other signal pathways related to glial production and renal development.

Through KEGG enrichment analysis of differential genes, we found that the Allen’s strike group was the most enriched in KEGG pathway terms on day 14 after SCI, with upregulated differential genes enriched in 95 KEGG pathway terms and downregulated differential genes enriched in 70 KEGG pathway terms. Upregulated KEGG pathways included “ECM-receptor interaction”, “NOD-like receptor signaling pathway”, “cytokine-cytokine receptor interaction”, “NF-kappa B signaling pathway”, “lysosome”, “osteoclast differentiation”, “protein digestion and absorption”, “Th17 cell differentiation”, “adipocytokine signaling pathway”, and “PI3K-Akt signaling pathway”. Downregulated KEGG pathways included “dopaminergic synapse”, “neuroactive ligand-receptor interaction”, “cAMP signaling pathway”, “insulin secretion”, “aldosterone synthesis and secretion”, “biosynthesis of amino acids”, “GABAergic synapse”, “GnRH secretion”, “inflammatory mediator regulation of TRP channels”, and “oocyte meiosis”. In contrast, in the complete paraplegia group, the upregulated differential genes were not enriched in any KEGG terms at 14 days after SCI, while there were three downregulated KEGG terms, which were “Cell cycle”, “Oocyte meiosis”, and “Progesterone-mediated oocyte maturation”. “Oocyte meiosis” was downregulated in both groups at 14 days after SCI. Previous studies have shown that the key activities that drive the process of meiosis are maturation-promoting factor, cyclin B, and a heterodimer of cell division cycle 2 kinase [33, 34].

Through the overlapping gene sets of the two SCI models, we can understand the common molecular characteristics of SCI. “L17 Signaling Pathway (RNO04657)”, “selenocompound metabolism (RNO00450)”, “non-small cell lung cancer (RNO05223)”, “melanoma (RNO05218)”, “bladder cancer (RNO05219)”, “EGFR tyrosine kinase inhibitor resistance (RNO01521)”,”adipocytokine signaling pathway (RNO04920)”, “sphingolipid signaling pathway (RNO04071)”, “MAPK signaling pathway (RNO04010)”, “estrogen signaling pathway (RNO04915)”, and “RAS signaling pathway (RNO04014)” were upregulated while “steroid hormone biosynthesis (RNO00140)”, and “oocyte meiosis (RNO04114)” were downregulated at 1 day post-injury. “lysine degradation (RNO00310)”, “renin angiotensin system (RNO04614)”, and “AMPK signaling pathway (RNO04152)” were upregulated and there was no overlapping downregulated gene set between the two SCI models at 3 day post-injury. “basal transcription factors (RNO03022)”, “ribosome biogenesis in eukaryotes (RNO03008)”, “RNA degradation (RNO03018)”, and “mRNA surveillance pathway (RNO03015)” were upregulated, whereas “steroid biosynthesis (RNO00100)” and “serotonergic synapse (RNO04726)” were downregulated at 7 day post-injury. “primary bile acid biosynthesis (RNO00120)”, “drug metabolism cytochrome P450 (RNO00982)”, “insulin resistance (RNO04931)”, “lysine degradation (RNO00310)”, and “chemical carcinogenesis (RNO05204)” were upregulated, whereas there was no overlapping downregulated gene set between the two SCI models at 14 day post-injury. “Starch and sucrose metabolism (RNO00500)” was upregulated and “steroid biosynthesis (RNO00100)” and “snare interactions in vesicular transport (RNO04130)” were downregulated at 56 day post-injury. “Lysine degradation (RNO00310)”, “circadian rhythm (RNO04710)”, “Phenylalanine metabolism (RNO00360)”, “longevity regulating pathway multiple species (RNO04213)”, “ABC transporters (RNO02010)”, “tyrosine metabolism (RNO00350)”, “AMPK signaling pathway (RNO04152)”, “renin secretion (RNO04924)”, and “purine metabolism (RNO00230)” were upregulated, whereas “fat digestion and absorption (RNO04975)” was downregulated at 70 day post-injury.

In summary, through RNA-seq technology and bioinformatics analysis, it was found that the transcriptomes of the two SCI models were different, and the differences were concentrated in the first 2 weeks after the SCI occurred. The main molecular events after SCI in the complete paraplegia group were more concentrated in the week after injury, reaching a peak on day 7. The major molecular events after SCI in the Allen’s strike group occurred immediately, but they were adjusted on the third day, and the duration was relatively long of up to 14 days. This study analyzed the changes in gene expression levels of different SCI models at different timepoints, investigated signal pathways enriched by different genes, elucidated molecular changes after SCI and SCI repair pathways, and further clarified the mechanism of SCI at the level of gene expression changes. Most early transcription events appear to be stabilized within 2 weeks after the injury, such that the overall change in the average gene expression was no longer obvious, and the expression level remained relatively stable. The downregulation of “Fat digestion and absorption” at 70 days after injury may be closely associated with the formation of glial scars after SCI. On the other hand, comparing and analyzing the difference in gene expression between complete paraplegia group and Allen’s group provides a reference for explaining the entire molecular process of SCI as well as a novel direction for spinal cord injury treatment.

## Conclusions

The transcriptomes of the two SCI models were different, and these differences were mainly concentrated in the first 2 weeks after the SCI. In the complete paraplegia group, the main molecular events after SCI were more concentrated during the first week after the injury, reaching a peak on day 7. Major molecular events after SCI in the Allen’s strike group lasted for a relatively long time, up 14 days. This study found that most of the early transcription events appeared to be stabilized within 2 weeks after injury, and the down-regulation of “fat digestion and absorption” at 70 days after injury may be closely associated with the formation of glial scars after SCI. These studies may provide some meaningful insights into the differences of targeted treatment strategies by explaining the entire molecular process after SCI.

## Data Availability

All data generated or analyzed during this study are included in this published article.
